# The Role of Healthcare Professionals’ Passion in Predicting Secondary Traumatic Stress and Posttraumatic Growth in the Face of COVID-19: A Longitudinal Approach

**DOI:** 10.3390/ijerph18094453

**Published:** 2021-04-22

**Authors:** Jennifer E. Moreno-Jiménez, Luis Manuel Blanco-Donoso, Evangelia Demerouti, Sylvia Belda Hofheinz, Mario Chico-Fernández, Bernardo Moreno-Jiménez, Eva Garrosa

**Affiliations:** 1 Faculty of Psychology, Universidad Autónoma de Madrid, 28049 Madrid, Spain; luismanuel.blanco@uam.es (L.M.B.-D.); bernardo.moreno@uam.es (B.M.-J.); eva.garrosa@uam.es (E.G.); 2 Human Performance Management Group, Eindhoven University of Technology, 5600 MB Eindhoven, The Netherlands; E.Demerouti@tue.nl; 3 Department of Industrial Psychology and People Management, University of Johannesburg, Johannesburg 2006, South Africa; 4 Intensive Medicine Service, Hospital Universitario 12 de Octubre de Madrid, 28041 Madrid, Spain; sylviabeldahofheinz@gmail.com (S.B.H.); murgchico@yahoo.es (M.C.-F.)

**Keywords:** COVID-19, healthcare professionals, passion for work, secondary traumatic stress, posttraumatic growth

## Abstract

COVID-19 has increased the likelihood of healthcare professionals suffering from Secondary Traumatic Stress (STS). However, the difficulty of this crisis may lead these professionals to display personal resources, such as harmonious passion, that could be involved in posttraumatic growth. The goal of this study is to examine the STS and posttraumatic growth among healthcare professionals and the demands and resources related to COVID-19. A longitudinal study was carried out in April 2020 (T1) and December 2020 (T2). The participants were 172 health professionals from different health institutions and they reported their workload, fear of contagion, lack of staff and personal protection equipment (PPE), harmonious passion, STS and posttraumatic growth. The results revealed that workload and fear of contagion in T2 were positive predictors for STS, whereas harmonious passion was a negative predictor. Fear of contagion of both times seemed to positively predict posttraumatic growth, as well as harmonious passion. One moderation effect was found concerning the lack of staff/PPE, as posttraumatic growth was higher when the workload was high, especially in those with a high lack of staff/PPE. All in all, these findings pointed out the need for preventative measures to protect these professionals from long-term negative consequences.

## 1. Introduction

Undoubtedly, the current crisis of the COVID-19 disease pandemic has strongly impacted on the general population, being declared as a Public Health Emergency of International Concern (PHEIC) [1]. However, this impact has been noticeably harder when it comes to healthcare professionals on the front line [2] from different health institutions, such as hospitals [3], health centers [4], and nursing homes [5]. Previous studies of the first wave of the pandemic have considered these three institutions to have high vulnerability due to exposure to death and high rates of infected patients [3,5]. Research focused on healthcare professionals in the first wave of the pandemic remarks on the high risks of these professionals for suffering from occupational negative consequences such as burnout and Secondary Traumatic Stress (hereinafter STS) [6,7,8]. Moreover, findings related to this topic have pointed out the long-term effects of this pandemic on healthcare professionals’ well-being [9], taking into account its length and the efforts maintained over time by these professionals. Hence, the main goal of this longitudinal study is to examine the long-term impact of the COVID-19 crisis on healthcare professionals from different health institutions in Spain. Therefore, this study strongly contributes to the current literature mainly in two ways: firstly, to surpass the cross-sectional studies implemented worldwide during this crisis [10], and secondly, to strengthen the empirical data previously obtained in the first wave and establish those positive personal resources such as passion for work that could have a beneficial impact on these professionals in the face of the second wave of the COVID-19 crisis. These findings seem relevant to continue improving preventative measures that protect those healthcare professionals from the long-term consequences of the crisis.

A report launched on the 11 May 2020 revealed the critical rate of 40,961 cases of healthcare professionals infected, with Spain the top country in the ranking rates of professionals infected [11]. These critical rates linked to the face of the pandemic on the frontline are linked to an increase in their job demands that have involved a new challenge for the health systems and exacerbated the occupational hazards that previously existed [6,7,8,9,12]. Specifically, these job demands are the substantial increase in their workload during their working times [13,14], linked to the fear of contagion [4,15], as they are a high-risk population for being infected. In this regard, these specific job demands related to COVID-19 and widely studied during the first wave of the pandemic (April 2020) have been associated with negative outcomes such as STS [16], defined as the stress resulting from helping or wanting to help a traumatized patient [17].

However, this pandemic not only has caused an increase in job demands for these professionals but also challenges due to the absence or the lack of job resources, for example, lack of staff, as many healthcare professionals were infected by the time that personal protection equipment (PPE) diminished [18,19]. Moreover, the crucial point is that this increase in job demands and the lack of job resources have been maintained over the period of one year [20]. This maintenance of high job demands involves the loss of energy and resources [21], which has compelled these professionals to develop/use some personal resources that may protect them against the impact of these job demands and the lack of job resources, in order to restore this energy loss [22,23]. Therefore, this study aims to study the role of passion for work as a personal resource in the face of the COVID-19 crisis.

The role of passion for work in healthcare professionals has arisen in recent years and constitutes a good preventative model in the field of burnout [24] and STS [25]. Passion for work has been defined as the inclination and love towards one’s work that is considered as pleasant and important, that people identify with and decide to invest a great amount of time and resources on [26,27]. The main characteristic of this passion for work is its impact on professionals’ well-being, depending on its type, taking the form of harmonious or obsessive passion. On the one hand, harmonious passion is that type that allows moving resources and making great investments on work while keeping a balance with other life’s activities, allowing to maintain harmony [28,29]. The harmoniously passionate worker enjoys their work and identifies with it but they are able to engage in other life activities, such as family or social ones [30]. This harmonious passion has been associated with job performance, life satisfaction, and less burnout [24,31]. On the other hand, obsessive passion involves a huge amount of time and effort in work, impeding a great balance between personal life and work. Although the negative consequence of obsessive passion still remains unclear [32,33], it has been generally related to more rumination, negative affect, and in turn, more burnout [34,35].

Concerning STS, new findings reveal that this passion for work, specifically harmonious passion, may protect people from STS, having a direct effect on the first hand, and may moderate the relationships between job demands and STS on the other hand. Indeed, those healthcare workers with high harmonious passion are those with less increase in STS when work stressors arise [25] and thus have a buffering role. These findings suggest that the role of harmonious passion as a protector seems undeniably relevant, buffering the impact of high demands maintained over time and preventing healthcare workers from negative consequences [36]. Furthermore, we aim to go a step further and examine whether harmonious passion changes trauma perception and is involved in positive outcomes after trauma.

Taking a closer look at the bright side of trauma reveals a positive experience that can happen after a crisis, i.e., posttraumatic growth. Posttraumatic growth has been defined as the positive changes and effort that an individual who suffers from a traumatic experience make in order to overcome this trauma and learn from it [37,38]. More specifically, when a traumatic experience is maintained over time, professionals may develop some coping skills that allow them to cognitively restructure this experience and in turn, lead to a positive outcome [39,40]. The literature supports that the development of these coping skills to produce cognitive changes is essential to allow this posttraumatic growth, and in some points could be strongly related to personality traits and social support [41]. Hence, as mentioned above, the COVID-19 outbreak has created a scenario with traumatic stimuli specifically for healthcare professionals, making them more likely to develop STS [40]. Despite this, the healthcare professionals that develop a harmonious passion profile make a great deal of effort to keep a balance with other life activities, avoiding conflict, guilt or another negative affect when they are unable to work [42]. Linked to that, harmoniously passionate workers may see their work environment as less harmful and seek more social support [43], experiencing more positive affect and emotions when they are carrying out their obligations at work [42]. These facts could lead to positive changes in their method of perceiving their work and in turn, play an outstanding role in posttraumatic growth development.

**Figure 1:**
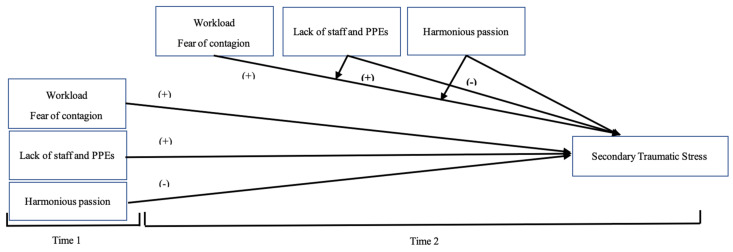
The proposed research model for predicting STS (Secondary Traumatic Stress) in the face of COVID-19.

**Figure 2:**
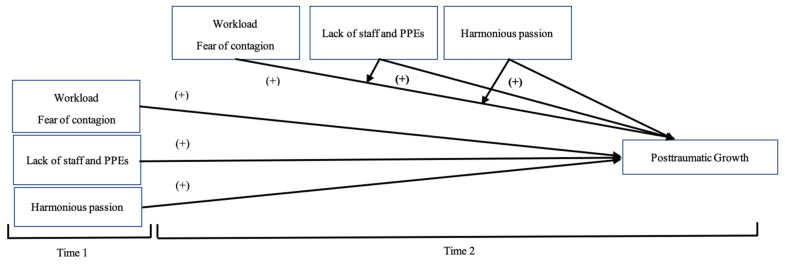
The proposed research model for predicting posttraumatic growth in the face of COVID-19.

All in all, the literature focused on the negative outcomes related to COVID-19 has grown in the last year, but to the best of our knowledge, very few studies examined the positive resources related to the resilience of the crisis, rather than being focused on negative outcomes [7]. This study tries to show how a negative experience of suffering from a crisis could lead to positive changes by experiencing this sense of growth. Moreover, even fewer studies have been carried out using a longitudinal approach [10,44], so we contribute by collecting data during the first wave of the pandemic (April 2020, hereinafter as T1) and the second wave (December 2020, hereinafter T2). Figure 1 and Figure 2 represent our theoretical research model that we aim to test. Hence, our hypotheses are the following:

**Hypothesis** **(H1).** *The demands related to COVID-19 (i.e., workload and fear of contagion) in T1 will positively predict (a) STS and (b) posttraumatic growth in T2.*

**Hypothesis** **(H2).** *The demands related to COVID-19 (i.e., workload and fear of contagion) in T2 will positively predict (a) STS and (b) posttraumatic growth in T2.*

**Hypothesis** **(H3).** *The lack of job resources related to COVID-19 in T1 (i.e., lack of staff and PPE) will positively predict STS (a) and negatively predict posttraumatic growth (b) in T2.*

**Hypothesis** **(H4).** *The lack of job resources related to COVID-19 in T2 (i.e., lack of staff and PPE) will positively predict STS (a) and negatively predict posttraumatic growth (b) in T2.*

**Hypothesis** **(H5).** *Harmonious passion in T1 will negatively predict (a) STS and positively predict (b) posttraumatic growth in T2.*

**Hypothesis** **(H6).** *Harmonious passion in T2 will negatively predict (a) STS and positively predict (b) posttraumatic growth in T2.*

**Hypothesis** **(H7).** *The lack of job resources (i.e., lack of staff and PPE) will have a moderating effect between the demands related to the COVID-19 (i.e., workload and fear of contagion) and (a) STS and (b) posttraumatic growth in T2. That is, when the staff and PPE resources are low, the increase in workload and fear of contagion will be related to a greater STS and posttraumatic growth.*

**Hypothesis** **(H8).** *Harmonious passion will have (a) a buffering effect between the demands related to the COVID-19 (i.e., workload and fear of contagion) and STS, and (b) a boosting effect between the demands related to the COVID-19 and posttraumatic growth in T2. That is, when harmonious passion is high, the increase in workload and fear of contagion leads to less STS and greater posttraumatic growth.*

## 2. Materials and Methods

### 2.1. Participants and Procedure

The participants of this study were 172 healthcare professionals. The participants belonged to different health institutions in Spain, from hospitals and healthcare centers (n = 64) on the one hand and nursing homes (n = 108) on the other hand. The total sample was composed of 83.1% females and 16.9% males, the mean age was 38.09 years and the average years of working experience was 17.92. The majority of the sample were nurses (27.0%) and nurse aides (26.7%), followed by physicians (20.3%). The sociodemographic and occupational data of the total sample are summarized in Table 1.

The data collected were part of a bigger project related to the impact of the COVID-19 crisis on health professionals [18]. During the first wave of the COVID-19 crisis (April 2020), an online questionnaire was created in order to assess the job demands, resources, and negative outcomes related to the development of this crisis in healthcare professionals. This online questionnaire was created through the Qualtrics online platform and the link was spread nationwide through social networks (such as Facebook, Instagram, Twitter and LinkedIn) and through direct contact with healthcare professionals who previously collaborated with the research team. Thus, the snowball technique was used to quickly spread the link (for a review of the procedure, see [18]). Then, at the end of the completion of this questionnaire, participants were asked about the possibility of participating in the follow-up of the study at the second phase (T2). They were asked to provide their email in order to get the link for the second phase (December 2020). In both phases, the participants received information about the study, the informed consent to be accepted and the information related to the voluntary nature of the study, allowing them the possibility to withdraw at their convenience. The participants were asked at the beginning of the questionnaire for some personal data (initial of your mother’s name, initial of your father’s name, day of your birth, the last two numbers of your birth year, and first two numbers of your mobile phone). This information allowed us to create a code, firstly to match the data from T1 and T2, and secondly to check whether they repeated the questionnaire completion, hence, this aspect was controlled. Within the total sample collected in T1 (N = 556), only 172 completed the T2, obtaining a response rate of 30.94%. Within the total sample (N = 556), only 208 healthcare professionals gave their informed consent to participate in the second phase, providing their email to be contacted later. Furthermore, this study obtained the approval of the Ethical Committee of Autonomous University of Madrid (CEI-106-2059).

### 2.2. Variables and Instruments

The following measures were repeatedly assessed two times as mentioned above, except for the variable posttraumatic growth as it only makes sense to be assessed after a crisis. Time 1 (T1) consisted of the data collected in April 2020 and Time 2 (T2) consisted of the data collected in December 2020. All measures showed good reliability indexes in both T1 and T2 (see Table 2). Due to the high workloads and pressures that specifically healthcare professionals are suffering from during this crisis, all the scales were shortened in order to reduce the length of the completion of the questionnaire and to facilitate the participation, taking a total of 10 min. The measures were the following:

Sociodemographic data such as sex, center, job position, years of work experience, and being in contact with a COVID-19 patient (see Table 1 and Table 2).

Workload. The perception of workload was assessed through the Spanish validation of the Secondary Traumatic Stress Scale (STSS) [45]. It is a five-item scale that evaluates the amount of work and time pressure that a professional may feel during their working hours as a healthcare professional. An example of an item is “In our service, the time pressure to attend a notice is very high”. The Likert response scale ranged from 1 (“totally disagree”) to 4 (“totally agree”).

Fear of contagion. For this measure, a 3-item scale was designed ad hoc in T1 to assess the healthcare professionals’ perception of fear concerning the COVID-19 disease, asking about their fear of being infected by the virus as well as infecting their relatives. An example item is “I have fear of being infected by the virus”. The Likert response scale ranged from 1 (“totally disagree”) to 4 (“totally agree”).

Lack of staff and PPE. Similarly, to the previous scale, a 2-item scale was designed ad hoc in T1 to assess the perceptions of the lack of human resources (i.e., staff) and material resources (i.e., personal protection equipment). An example item is “The lack of individual protection equipment scares me”. The Likert response scale ranged from 1 (“totally disagree”) to 4 (“totally agree”).

Harmonious passion. A 3-item scale of the Spanish validation of “Passion toward Work Scale” was used [46]. This scale assesses the healthcare professionals’ passion toward their work, as they feel fulfilled by their work, identify themselves and keep a balance between work and other life domains. An example item is “My work is in harmony with my other life activities”. The Likert response scale ranged from 1 (“totally disagree”) to 7 (“totally agree”).

STS. This variable was measured using the previous STSS scale [45]. The scale consists of 14 items that assess the negative consequences and the cost of being exposed to traumatic stimuli as a helping professional. It assessed emotional fatigue (”I feel emotionally without strength”), shattered assumptions (“this work makes me see the world as unfair”), and symptomatology (“I keep vivid images about those accidents that affect me a lot”). For this study, the general measure of STS was used to assess the complete experience of secondary traumatic stress. The Likert response scale ranged from 1 (“totally disagree”) to 4 (“totally agree”).

Posttraumatic growth. This variable was assessed exclusively in T2. The Spanish adaptation of the Posttraumatic Growth Inventory-Short Form was used [37]. This scale consists of 10 items aimed at assessing the sense of learning and coping after a crisis experience, making positive changes as an effort to process a traumatic experience. This posttraumatic growth includes dimensions of new possibilities, relating to others, personal strength, appreciation of life, and spiritual change. An example item is “I changed my priorities about what is important in life”. The Likert response scale ranged from 1 (“completely in disagreement”) to 6 (“completely in agreement”).

### 2.3. Statistical Analysis

Firstly, means, standard deviations, and Pearson correlations were carried out to investigate the descriptive data of the sample, both in Time 1 and Time 2 (see Table 2). Secondly, a t-test for paired samples was used to see whether there were significant differences concerning the demands (i.e., workload and fear of contagion), job resources (i.e., lack of staff and PPE), personal resources (i.e., harmonious passion) and the outcome (i.e., STS) in T1 and T2. Thirdly, Student’s t-test for independent samples was run as well to test possible differences concerning sex and center (see Table 3) and a one-way ANOVA compared groups to check this depending on job position (showed as an additional analysis). Finally, to test our main hypothesis we conducted hierarchical multiple regression using stepwise regression (see Table 4).

For this purpose, the outcomes of our models were STS (model 1) and Posttraumatic Growth (model 2) in T2, that act as criteria variables. The total model was composed of four steps as follows. The first step includes the control variables such as sex and center, as sex has shown to have a differential role in the face of COVID-19 [47], and center displayed significant differences in our sample. Moreover, in the STS model (model 1), STS in T1 was included as a control of the accumulative effect of this time in the STS in T2. In the case of the posttraumatic growth model (model 2), we could not establish as control T1 as this variable can only be assessed after a period of time from the beginning of a crisis, as it means to make changes in order to overcome the traumatic experience. The second step included the variables assessed in T1 (workload, fear of contagion, lack of staff and PPE and harmonious passion). The third step included the same variables but in T2. The fourth step included the moderation analysis of the demands (workload and fear of contagion) with the resources (lack of staff and PPE and harmonious passion) in predicting both outcomes (STS and posttraumatic growth in T2), all of them in T2. All variables were centered to avoid the problem of multicollinearity. Finally, to test the significance of the interaction effect, a simple slope test was conducted following the criteria of Preacher, Curran and Baue [48]. All data were analyzed using SPSS 26.0 program (IBM, Chicago, IL, USA).

## 3. Results

### 3.1. Preliminary Analysis

Prior to test our main hypothesis, the means, standard deviations, and bivariate correlations among the interested variables were calculated (see Table 2). As our interest was to observe the evolution of demands (i.e., workload and fear of contagion), resources (i.e., lack of staff and PPE and harmonious passion), and outcomes during these two waves of the pandemic (i.e., STS and posttraumatic growth), we tested whether there were significant differences in these two times in the different variables. Table 3 shows this Student’s t-test for paired variables, obtaining the mean differences.

First, starting with the variable time, as observed, the workload presents neither an increase nor a reduction in its levels concerning the first and second waves. On the other hand, an interesting result came from the fear of contagion and the lack of staff and PPE in the second wave, presenting a significant reduction in their levels in comparison with their levels in the first wave (*t* = −3.007; 95% CI [−0.298, −0.061]; *p* < 0.01*,* and *t* = 6.414; 95% CI [−0.596, −0.315]; *p* < 0.001, respectively). The harmonious passion maintained high levels in both times, finding no significant difference. Finally, STS experienced an increase in its levels in T2 in comparison with T1 (*t* = 2.439*;* 95% CI [0.014, 0.13]; *p* < 0.01).

Second, sex and center differences were calculated to establish whether these variables play an important role (Table 3). Regarding the variable sex, we could find significant differences in: workload of T2, being higher in females ($\bar{X}=3.229;\;$*t* = −2.559; 95% CI [−0.455, −0.058]; *p* < 0.01); fear of contagion of T2, being higher in females ($\bar{X}=2.617;\;$*t* = −2.178; 95% CI [−0.592, −0.022]; *p* < 0.05); and STS, being higher in females as well ($\bar{X}=2.818;\;$*t* = −2.671; 95% CI [−0.358, −0.087]; *p* < 0.01). Concerning the variable center, we only found significant differences in posttraumatic growth, which was higher in nursing homes ($\bar{X}=4.307;\;$*t* = −3.834; 95% CI [−0.850, −0.269] *p* < 0.001). Third, additional analysis through ANOVA were conducted to compare groups with different job positions and this revealed a significant difference between (a) physicians and (b) nurse aides concerning the lack of resources and posttraumatic growth, being higher in nurse aides ($\bar{X}{}_{a-b}=-0.610;\;$*F* = 3.017; 95% CI [−1.15, −0.069]; *p* < 0.05, and $\bar{X}{}_{a-b}=-0.808;\;$*F* = 4.889; 95% CI [−1.34, −0.277]; *p* < 0.001).

### 3.2. Hypothesis Testing

Table 4 shows the demands and resources related to COVID-19 and harmonious passion in predicting STS (model 1) and posttraumatic growth (model 2). Starting with STS (model 1), we take a closer look at the percentage of explained variance. As observed in Table 4, the first and third steps are the ones that involve a significant increase in the explained variance, resulting in significance (*R^2^ =* 0.32, and *R^2^ =* 0.49, respectively). In this sense, the inclusion of the control variables, especially STS in Time 1, and the demands and resources in T2, were the variables that better explained the levels of STS in T2. Concretely, none of the demands of T1 resulted in being positive predictors of STS, so we did not confirm H1(a). In contrast, only STS in T1 was a positive predictor (*B* = 0.410; *p* < 0.001), followed by the workload (*B* = 0.203; *p* < 0.01) and fear of contagion (*B* = 0.187; *p* < 0.01) in T2. Thus, we found statistical support only for H2(a) concerning the demands. In regard to the resources, we did not find any statistical support for lack of staff and PPE as a positive predictor of STS, so we did not support H3 and H4(a), or the interaction effect as hypothesized in H7(a). However, harmonious passion in Time 2 seems a negative predictor of STS, thus confirming H6(a). No statistical support was found for the moderator role of harmonious passion in STS i.e., for H8(a).

Concerning the posttraumatic growth (model 2), we observed a significant increase in the second, third, and fourth steps, having a greater increase in the third step with the inclusion of the variables related to T2 ($\Delta R^{2}=0.05;p<0.01).$ The final step and the total model allowed us to explain 22% of the variance. In this regard, the center is the only positive predictor of the control variable, as we previously explained in Table 4 that significant differences were found depending on the center (*B* = 0.399; *p* < 0.001). We did not find statistical support for H1(b) despite fear of contagion being significant until the interaction effect was added. Linked to that, fear of contagion in T2 seems a positive predictor for posttraumatic growth **(***B* = 0.241; *p* < 0.01) and thus partially confirmed H2(b). Regarding resources, only harmonious passion in T2 positively predicted posttraumatic growth (*B* = 0.210; *p* < 0.05), finding statistical support only for H6(b).

### 3.3. Interaction Effects

**Figure 3:**
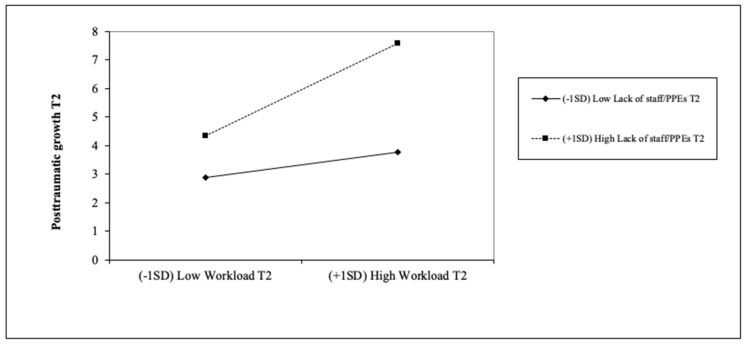
Interaction effects of workload and lack of staff/PPE in T2 in predicting posttraumatic growth in T2. *Note*: PPE = Personal Protection Equipment; T2 = second wave of COVID-19 (December 2020).

As mentioned above, no interaction effect was found concerning STS in T2 (model 1). Therefore, no statistical support was found for H7(a) and H8(a) (see Table 4). In contrast, despite the fourth step of posttraumatic growth resulted in not being significant as well, due to the small increase in the explained variance of posttraumatic growth (step 3, model 2), we found a significant interaction effect that seems interesting to comment on. Specifically, we observed a significant interaction effect concerning the lack of staff and PPE and workload in predicting posttraumatic growth (see Figure 3). A simple slope test was conducted following the advice of Preacher, Curran and Baue [48] and we found this interaction is significant specifically for those with high levels of lack of staff/PPE. Indeed, workload is positively related to posttraumatic growth, as this seems to increase when passing from low to high workload. Moreover, this increase is much greater when healthcare professionals perceived a high lack of staff/PPE (gradient of slope = 1.336, *t* = 1.998, *p* < 0.05), providing support for H7(b). However, as this step was not significant, posttraumatic growth was better explained through the predictive values of the variables of T2 in this second wave.

## 4. Discussion

This study aimed to test the impact of the COVID-19 pandemic on healthcare professionals, providing data about the positive and negative predictors of STS and posttraumatic growth from a longitudinal approach. As shown, we aimed to test whether the presence of high job demands, such as workload and fear of contagion, and the absence of job resources, such as the lack of staff and PPE, in both the first and second wave, have as a negative consequence higher levels of STS. Moreover, this study includes the examination of the role of harmonious passion as a robust protector of STS, assessing its protecting effect in the long-term. Furthermore, our last goal was not only to study the evolution of such a relevant psychosocial risk as STS but also the positive side after a crisis through posttraumatic growth. All in all, we further contribute to the current literature by providing empirical evidence about all these questions, surpassing the previous literature focused on cross-sectional designs [10].

Firstly, as was supposed, the previous level of STS in T1 (first wave of the crisis) resulted in a strong positive predictor for STS in T2 (second wave of the crisis). Thus, once the STS has been developed in the first wave, it seems to continue growing, possibly due to the accumulative effect of the job demands being maintained. In this sense, both workload and fear of contagion, as the job demands related to the COVID-19 crisis, positively and significatively predict STS in the second wave, as previously found [6]. Thus, the constant exposure to death and suffering and the fear of being infected, as specific characteristics of this pandemic, mean that these healthcare professionals have been constantly exposed to traumatic stimuli, and consequently are more likely to develop this STS, as authors have pointed out [7].

Despite this, data collected in the second wave revealed that workload seems to be stable with no significant differences between the first and the second wave (maintaining high levels), whereas there is a significant decrease in the fear of contagion. This diminishment could be related to the decrease in the lack of staff and PPE, as previous findings revealed that this lack leads to more fear of contagion [49,50]. Specifically, research carried out in the first wave of COVID-19 in April 2020 revealed that the lack of resources, such as personal resources and PPE, leads to an increase in workload and fear of contagion, and in turn, this is associated with more STS [18].

On a positive note, as previous studies confirmed, harmonious passion seems to play a protector role in STS development, which could be explained by the recovery activities and the strong balance of these harmoniously passionate workers [24,28]. Indeed, the data revealed that healthcare professionals are facing hard working conditions on the frontline, with high levels of workload (maintained over time), and fear of contagion, which may lead, in turn, to developing/using harmonious passion as a way to allow recovery after work and diminish the negative impact of these job demands. In this line of evidence, authors have remarked on the need to use positive resources that allow recovery and buffer the effects of demands on professionals’ well-being [21]. Moreover, these findings are supported by previous studies that revealed the use of stable resources when job demands become more stressful to avoid fatigue [22]. In fact, these statements could be an explanation of the high levels of harmonious passion maintained over time in the two waves, as a positive and stable resource to keep optimum levels of well-being. Looking closely, previous findings revealed that this harmonious passion prevents compassion fatigue, as the emotional fatigue related to STS [25], that implies the effect of this passion in the emotional component. Linked to that, these findings reveal as well how this harmonious passion could change the relationship of the professional with the job demands, as previous authors confirmed [43]. No interaction effect was found concerning STS, thus, we need to continue improving the variables studied to check those resources with greater impact. This means that harmonious passion cannot particularly protect employees that are confronted with high job demands during the pandemic, and previous studies have found this protector role when job demands are high if we consider the cognitive changes of the trauma, that is, shattered assumptions [25]. Possibly, harmoniously passionate workers are able to see less overload and more job control during their working times [43], but the excessive increase of job demands during this pandemic makes it difficult to moderate this relationship. These findings pointed out the need to improve the hard job demands that these healthcare professionals are facing, that are directly related to STS development [14].

Secondly, our need to pay attention to the bright side of the trauma revealed interesting results. On one hand, the demands related to the traumatic stimuli, that is, fear of contagion, were positively and significantly related to posttraumatic growth in this second wave. These data show that the high load of these specific traumatic demands on the frontline may predict a posttraumatic growth in the medium term, especially when these traumatic demands are a bit lower (as occurs with fear of contagion in the second wave), possibly making the healthcare professionals more aware of it and allowing cognitive changes [39,40]. As an interesting result, workload resulted in a not significant predictor for this variable. In this regard, workload seems to relate to the development of negative psychosocial risks [21] but not to the positive experience of growth after trauma. On the other hand, the outstanding role of harmonious passion seems relevant as well in predicting posttraumatic growth. In this sense, harmonious passion positively and significantly predicts this posttraumatic growth, as it is possibly associated with positive cognitive changes and a positive vision of the work setting, despite the job demands [42,43]. In this regard, the ability to keep a balance between work and other life areas despite the feeling of being overwhelmed by work may display cognitive changes that allow healthcare professionals to be engaged with more recovery experiences after work [51], facilitating less STS and posttraumatic growth. This passion may constitute a way to disengage from their hard work on the frontline, allowing more recovery experiences after work [24], seeking social support, considered as protective [14], and boosting cognitive changes in their values. Consequently, these attitudes after work may enhance others life activities [34]. Furthermore, this harmonious passion has been related to the use of strengths, both being positive predictors of well-being [36] and possibly related to posttraumatic growth. More specifically, harmonious passion could be considered as a personality trait that may play an outstanding role in this posttraumatic growth [41], as it seems to be stable over time in the two waves, maintaining high levels. Moreover, further studies about harmonious passion’s role in posttraumatic growth are needed to explain such a process.

However, despite the role of fear of contagion in both waves as well as harmonious passion in the second wave strongly and positively predicting this posttraumatic growth, an interesting interaction effect was found. Specifically, it seems that when the workload is increasing, those with a higher lack of staff and PPE experienced more posttraumatic growth. These data revealed the critical scenario that healthcare professionals are facing and confirmed the resilience theories about growth after trauma [38]. The differences found concerning the job position seem to support this interaction, as nurse aides suffering from a higher lack of staff and PPE experience higher posttraumatic growth.

Finally, our findings revealed non-significant differences concerning the center (hospitals/health centers and nursing homes) except for posttraumatic growth, which was higher in nursing homes. One reason could be the high pressure and workload that they could have suffered from the beginning of the pandemic, as other studies have pointed out the impact of the COVID-19 outbreak on some private health sectors, where the lack of information and protocols necessary to overcome the initial impact may increase ambiguity and distress [52]. Due to the initial hazards that they may face, as they were considered in the first wave as a high-vulnerability sector [5], nursing homes could have started in the second wave experiencing this posttraumatic growth once the resources were stable and protocols were well established, showing higher levels in comparison with hospitals and health centers. Similar to this, the differences found between physicians and nurse aides may confirm this tentative explanation, as the higher the lack of resources, the higher the posttraumatic growth, possibly as a way to overcome this initial trauma and the lack of clear procedures [38]. Interestingly, previous studies have pointed out that being female (as the majority of nurse aides are) and the lack of resources are risk factors for developing STS [14,53], which was confirmed by our findings as well since females presented higher STS. Furthermore, in our case and possibly due to the temporality, the presence of job demands seems to have a huge impact on its developments, prior to sex and job resources. The study carried out by Pappa et al. [54] suggests the caution needed to interpret all COVID-19 studies due to their heterogeneity, methodological issues (e.g., different scales to assess STS), and time frames.

Interestingly, this second wave could be the starting point to examine the evolution of this posttraumatic growth, and the variables more related to this development. As data revealed, the job demands continue to be high, as well as the lack of job resources. At this point, both factors could enhance the initial posttraumatic growth of the crisis, but there could be other variables that help professionals to cope with the trauma, such as social support, which may play an important role as well [41].

## 5. Conclusions

Public health services may take preventative measures to prevent the long-term effects of this pandemic on professionals, as many studies have pointed out [15]. Specifically, the work environment within these health institutions could promote and give an important space to harmonious passion, giving the professionals time for recovering and emphasizing the importance of self-care. As this harmonious passion seems to create positive changes in the professionals, it may allow them to see the actual crisis as an opportunity to grow and learn, making them feel more capable to overcome the job demands and, in turn, suffer less from STS. All in all, health institutions may boost these perceptions and facilitate its integration, for example, through reinforcing the possibility to have more free days and giving psychoeducational workshops about (a) the psychosocial risks and the stressors associated with their development, (b) how to identify stressors to individually apply strategies to overcome them and (c) remarking on the importance of recovery time, providing strategies for disengaging from work and enhancing the need to pursue personal values. These intervention measures may be a way to reduce healthcare professionals’ vulnerability, which is undeniably relevant in this crisis time [4,14].

## 6. Limitations

This study has some limitations that deserve to be mentioned. Firstly, as the data collected were part of a bigger project, two samples were analyzed that could alter the results obtained. As the two samples were not big enough, we could not display statistical analyses separately. In spite of this issue, we aimed to control this effect by adding “center” as a control variable and observing possible differences. Thus, future research could consider analyzing the STS and posttraumatic growth considering the health institution. Secondly, as the healthcare professionals were suffering from overload at work, we aimed to reduce the length of the questionnaire by selecting the most significant items of each variable. This procedure may have led to the fact that the reliabilities of some scales (i.e., harmonious passion) are lower than expected. Finally, as no moderation effect was found, we need to continue improving the theoretical model to achieve a better understanding of STS and posttraumatic growth in this crisis by adding more variables related to job resources, such as co-worker and supervisor support.

## Figures and Tables

**Table 1:** Sociodemographic and occupational data of the total sample.

Sociodemographic and Occupational Data	Total Health Professionals (*N* = 172)	
Categorical sociodemographic variables	*n*	%
Sex		
Male	29	16.9
Female	143	83.1
Sentimental Relationship		
With a relationship	132	76.7
Without a relationship	40	23.3
Quantitative sociodemographic variables	*M*	*SD*
Age	38.09	10.67
Job Position		
Physician	35	20.3
Nurse	48	27.9
Nurse aides	46	26.7
Occupational therapist	5	2.9
Psychologist	16	9.3
Social workers	13	7.6
Physiotherapist	9	5.2
Categorical occupational variables		
Centre		
Hospitals and health centers	64	37.2%
Nursing homes	108	62.8%
Contact with COVID-19 patient		
Yes	57	33.1
No	7	4.1
Missing Values	64	62.8
Quantitative occupational variables		
Years of experience in the field	17.92	11.48

**Table 2:** Means, standard deviations, internal consistency indexes (Cronbach’s alpha) and bivariate correlations.

Variables	$\bar{X}$	SD	α	1	2	3	4	5	6	7	8	9	10	11
1. Workload T1 ^a^	3.13	0.49	0.80	-	0.27 **	0.41 **	−0.01	0.42 **	0.33**	0.03	0.17 *	−0.16 *	0.29 **	−0.03
2. Fear of contagion T1 ^a^	2.74	0.76	0.80		-	0.49 **	−0.05	0.37 **	0. 16 **	0.46 **	0.24 **	0.05	0.24 **	0.17 **
3. Lack of staff and PPE T1 ^a^	3.16	0.73	0.68			-	−0.09 *	0.45 **	0.11	0.31**	0.29 **	−0.19 **	0.32 **	0.05
4. Harmonious passion T1 ^b^	5.09	1.20	0.68				-	−0.22 **	−0.04	−0.02	−0.07	0.49 **	−0.32 **	−0.08
5. STS T1 ^a^	2.70	0.45	0.84					-	0.24 **	0.12	0.19 *	−0.26 **	0.58 **	0.04
6. Workload T2 ^a^	3.18	0.50	0.73						-	0.17 *	0.27 **	−0.11	0.41 **	0.04
7. Fear of contagion T2 ^a^	2.56	0.76	0.80							-	0.37 **	−0.07	0.33 **	0.19 *
8. Lack of staff and PPE T2 ^a^	2.70	0.83	0.72								-	−0.12	0.36 **	0.08
9. Harmonious passion T2 ^b^	5.07	1.17	0.69									-	−0.38 **	0.14
10. STS T2 ^a^	2.78	0.42	0.82										-	0.12
11. Posttraumatic growth T2 ^c^	4.11	0.84	0.80											-

*Note*: PPE = Personal Protection Equipment; STS = Secondary Traumatic Stress; T1 = first wave of COVID-19 (April 2020); T2 = second wave of COVID-19 (December 2020). ^a^ the scale response ranged from 1 to 4; ^b^ the scale response ranged from 1 to 7; ^c^ the scale response ranged from 1 to 6; ** *p* < 0.01; * *p* < 0.05.

**Table 3:** Significant differences among the interested variables concerning time (the first and the second wave of COVID-19 crisis), sex and center.

Variables	Time		Sex			Centre		
	$\bar{X}$ ^T2^ $-\bar{X}$ ^T1^	*t*	$\bar{X}$ ^males^	$\bar{X}$ ^females^	*t*	$\bar{X}$ ^H&HC^	$\bar{X}$ ^NH^	*t*
1. Workload ^a^	0.053	1.228	2.972	3.229	−2.559 **	3.137	3.215	−0.958
2. Fear of contagion ^a^	−0.180	−3.007 **	2.310	2.617	−2.178 *	2.635	2.524	0.923
3. Lack of staff and PPE ^a^	−0.456	−6.414 ***	2.500	2.741	−1.450	2.570	2.777	−1.625
4. Harmonious passion ^b^	−0.022	−0.237	4.942	5.098	−0.783	5.250	4.972	1.451
5. STS ^a^	0.074	2.439 **	2.595	2.818	−2.671 **	2.699	2.827	−1.813
6. Posttraumatic Growth	−	−	4.155	4.095	0.416	3.747	4.307	−3.834 ***

*Note*: PPE = Personal Protection Equipment; STS = Secondary Traumatic Stress; T1 = first wave of COVID-19 (April 2020); T2 = second wave of COVID-19 (December 2020); H&HC = Hospitals and Health Centers; NH = Nursing Homes; ^a^ the scale response ranged from 1 to 4; ^b^ the scale response ranged from 1 to 7. Sex was codified as 0 = males and 1 = females; Centre was codified as 1 = hospital and health centers and 2 = nursing homes; *** *p* < 0.001; ** *p* < 0.01; * *p* < 0.05.

**Table 4:** Hierarchical regression model on criterion variables of Secondary Traumatic Stress and Posttraumatic Growth.

Predictors	Model 1				Model 2			
	Secondary Traumatic Stress				Posttraumatic Growth			
Step 1: Control	Standardized β				Standardized β			
Sex	0.084	0.106	0.050	0.039	−0.054	−0.069	−0.103	−0.069
Centre	0.091	0.067	0.051	0.055	0.349 ***	0.361 ***	0.389 ***	0.399 ***
STS T1	0.534 ***	0.490 ***	0.402 ***	0.410 ***	−	−	−	−
Step 2: variables T1								
Workload		0.054	−0.001	−0.009		−0.065	−0.089	−0.023
Fear contagion		0.081	0.038	0.057		0.183 *	0.214 **	0.182
Lack staff/PPE		−0.012	0.013	0.002		−0.145	−0.109	−0.089
Harmonious passion		−0.123	−0.044	−0.042		0.159 *	0.065	0.054
Step 3: variables T2								
Workload			0.203 **	0.207 **			0.007	0.027
Fear contagion			0.187 **	0.192 **			0.247 **	0.241 **
Lack staff/PPE			0.130 *	0.119			−0.053	−0.026
Harmonious passion			−0.195 **	−0.192 **			0.191 *	0.210 *
Step 4: Moderations T2								
Workload × Lack staff/PPE				0.017				0.184 *
Fear contagion × Lack staff/PPE				0.066				−0.117
Workload × harmonious passion				0.046				−0.077
Fear contagion × harmonious passion				−0.041				0.084
$R^{2}$	0.32	0.33	0.49	0.46	0.11	0.15	0.20	0.22
$\Delta R^{2}$	0.32 ***	0.01	0.16 ***	−0.03	0.11 ***	0.04 *	0.05 **	0.02

*Note:* STS = Secondary Traumatic Stress; PPE = Personal Protection Equipment; T1 = first wave of COVID-19 (April 2020); T2 = second wave of COVID-19 (December 2020); Sex was codified as 0 = male and 1 = female; Centre was codified as 1 = hospital and health centers and 2 = nursing homes; *R^2^* = Percentage of explained variance by the inclusion of variables; *** *p* < 0.001; ** *p* < 0.01; * *p* < 0.05.

## Data Availability

The data presented in this study are available on request from the corresponding author.
